# Analyzing Knowledge Retrieval Impairments Associated with Alzheimer’s Disease Using Network Analyses

**DOI:** 10.1155/2019/4203158

**Published:** 2019-05-02

**Authors:** Jeffrey C. Zemla, Joseph L. Austerweil

**Affiliations:** Department of Psychology, University of Wisconsin-Madison, USA

## Abstract

A defining characteristic of Alzheimer’s disease is difficulty in retrieving semantic memories, or memories encoding facts and knowledge. While it has been suggested that this impairment is caused by a degradation of the semantic store, the precise ways in which the semantic store is degraded are not well understood. Using a longitudinal corpus of semantic fluency data (listing of items in a category), we derive semantic network representations of patients with Alzheimer’s disease and of healthy controls. We contrast our network-based approach with analyzing fluency data with the standard method of counting the total number of items and perseverations in fluency data. We find that the networks of Alzheimer’s patients are more connected and that those connections are more randomly distributed than the connections in networks of healthy individuals. These results suggest that the semantic memory impairment of Alzheimer’s patients can be modeled through the inclusion of spurious associations between unrelated concepts in the semantic store. We also find that information from our network analysis of fluency data improves prediction of patient diagnosis compared to traditional measures of the semantic fluency task.

## 1. Introduction

Alzheimer’s disease (AD) is a debilitating neurodegenerative disease that affects roughly 46 million people worldwide [1]. A defining characteristic of AD is an increased difficulty in retrieving semantic memories (i.e., declarative knowledge of facts and concepts). Patients with AD have difficulty naming objects [2], matching semantically related pictures [3], and identifying the semantic features of words [4]. Though there are many neuropsychological tests for measuring semantic impairment due to AD, the mechanisms producing these deficits have not been isolated. While some research suggests these deficits are due to a degradation of a semantic memory store that encodes concepts in the mind [5–7], other evidence points to difficulty in retrieving memories from an intact semantic memory store [8–10].

One difficulty in resolving this debate is that the semantic memory store is not directly observable. To address this, computational models have been developed to explain how semantic memories are represented and to make inferences about the underlying mechanisms responsible for memory retrieval [11]. However most techniques for estimating semantic representations assume a common knowledge representation for a group, including patient populations [5, 12]. This is problematic for analyzing individuals with AD as their impairments tend to be heterogeneous [13, 14]. Aggregating over retrieval data of many patients to estimate a single group-based representation may result in an estimated representation that does not actually resemble any individual in the population [15, 16]. In this article, we use semantic networks as a model of how memories of facts and knowledge are encoded, develop a method for estimating networks from memory retrieval data, and use it to analyze data from individuals with AD and healthy controls at individual and cross-sectional levels.

There is a long history of modeling semantic knowledge using a semantic network [17, 18], an abstract representation of how concepts are organized in the mind. In a semantic network, concepts are represented by nodes and semantic similarity is represented by edges that connect pairs of nodes. In recent years, advances in network science have improved our understanding of semantic memory by providing us tools to quantify how networks are organized [19], and as a result semantic networks have been used to explore how conceptual knowledge is affected by factors such as creativity [20], bilingualism [21], age [22], and more.

Among the most commonly used tasks to diagnose semantic memory impairment is the semantic fluency task [23], in which participants list as many items from a category (e.g., animals) as they can in a short period of time (e.g., one to three minutes). This test is part of several popular neuropsychological batteries, including the Cognitive Linguistic Quick Test [24] and Uniform Data Set [25]. Traditionally, the fluency task is scored by counting the number of perseverations (repetitions) listed by a participant, as well as the number of items listed, excluding perseverations and errors (responses not in the target category). Compared to healthy controls, individuals with AD routinely list fewer items [26] and have higher perseveration rates [27]. Even in presymptomatic individuals, perseverations in the fluency task have been associated with future cognitive decline [28], and the number of items listed has been associated with pathological markers of AD [29].

However semantic fluency data can also be used to estimate semantic networks of groups or individuals [30, 31]. This is possible because semantic fluency data tend to be clustered [32]: individuals often list multiple semantically related responses in sequence. For instance, when listing animals, a participant may list a sequence of pets (such as *dog, cat,* and *hamster)* before switching to a new cluster (e.g., zoo animals such as *giraffe, lion,* and *hippo).* Because semantically related words typically appear near each other in a fluency list, a semantic network of word associations can be estimated from a corpus of fluency data.

In this article, we apply a random walk model of semantic memory retrieval [31, 33] to a longitudinal dataset of semantic fluency data from AD patients and healthy controls in order to estimate semantic network representations of individuals and investigate mechanisms responsible for impaired performance due to AD. We compare these representations and find systematic differences in the structure of semantic representations between AD patients and control participants. This network-based analysis of semantic fluency data provides additional insight into the specific cognitive mechanisms that lead to memory impairments by identifying associations between properties of an individual’s semantic network and impaired behavioral performance.

To model impaired performance, we extend the random walk model of semantic memory retrieval [33] to account for perseverations in semantic fluency data. Perseverations in semantic fluency data are modeled as errors resulting from a faulty monitoring process associated with working memory, which is in line with current neuropsychiatric research [34]. We propose a generative computational model that accounts for perseverations in fluency data and demonstrate that it can quantitatively capture the severity of impairment to this monitoring process.

## 2. Materials and Methods

### 2.1. Participants and Design.

We acquired a longitudinal corpus of semantic fluency data from the University of California-San Diego Shiley-Marcos Alzheimer’s Disease Research Center (ADRC). Data were collected between 1985 and 2016 as part of the ADRC’s broader goal to better understand Alzheimer’s disease. The fluency data used for our analyses partially overlaps with data presented in previous publications from this ADRC (e.g., [35]).

Each participant visited the lab approximately once per year for the duration of their involvement and was tested on the semantic fluency task as part of a longer neuropsychiatric exam. The average length of participation was approximately 9 years per participant (i.e., most participants began after 1985 and/or discontinued participation prior to 2016.) Participants included healthy individuals, as well as those who were already diagnosed with AD or other memory-related issues. At each visit, patients were given a clinical diagnosis using the National Institute of Neurological and Communicative Disorders and Stroke-Alzheimer’s Disease and Related Disorders Association (NINCDS-ADRDA) scale [36]. This evaluation was based on multiple sources, including the participant’s performance on the Mini-Mental State Exam [37]. Of interest, these diagnoses included “Normal Control” (NC) and “Probable AD” (PAD), but a much smaller number of participants were given other diagnoses (such as “Mild Cognitive Impairment” or “Frontotemporal dementia”). To focus our analyses on AD and not related dementias, we limit our analysis of the dataset only to visits in which a participant was diagnosed as NC or PAD; all other visits were excluded. Diagnoses of AD using the NINCDS-ADRDA scale have been found to have good sensitivity and specificity when compared to postmortem pathological reports [38]; however, the scale is limited in that it does not account for secondary diagnoses (e.g., some patients diagnosed as PAD may also have pathological signs of Lewy body dementia). Newer clinical definitions of Alzheimer’s have evolved over time, most notably making use of in vivo biomarker detection for diagnosis (e.g., [39]). However due to the longitudinal nature of the dataset, a fixed classification scheme was used.

The animal semantic fluency task lasted one minute per visit. Participants named animals aloud, which were written down in real time on paper by a researcher conducting the task. In total, we transcribed 1,047 animal fluency lists generated from 123 participants (60% female, mean age at first visit 71.4, range 34–90). This set of participants excludes any individual who did not have at least three fluency lists when diagnosed as NC or three fluency lists when diagnosed as PAD. These lists are nonoverlapping. For example, a participant with 2 NC and 2 PAD visits was rejected outright (not included in the 123 participants). A participant with 3 NC and 1 PAD visit would have their NC visits analyzed, but not their PAD visits. Of those, 248 lists were generated from 41 participants who were diagnosed as PAD for that visit (51% female, mean age 75.2, range 61–90), while 799 lists were generated from 84 participants who were diagnosed as NC for that visit (64% female, mean age 69.5, range 34–86). These participant pools overlapped—2 participants were diagnosed as both NC and PAD (on separate visits).

### 2.2. Network Estimation Model.

**FIGURE 1:**
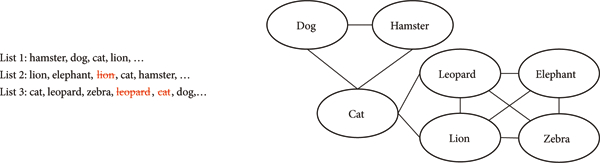
Semantic fluency lists (left) can be modeled as a censored random walk on a semantic network (right). When *p*_*emit*_ = 0, repeated items are “censored” on subsequent traversals, as shown above. When 0 < *p*_*emit*_ < 1 this censoring process is stochastic. Figure reprinted from Zemla and Austerweil [31] with permission from Springer.

As suggested by current best practices for estimating undirected, unweighted semantic networks from fluency data [31], we used U-INVITE to infer networks for each individual from their fluency data. U-INVITE is a method for estimating networks which assumes fluency data are generated by a censored random walk on that network [33,40]. Assuming an individuals fluency data are generated by a censored random walk, U-INVITE uses Bayesian inference to estimate the most likely network. In a censored random walk, states in the walk are observed when they are traversed for the first time, but are “censored” (unobserved) on subsequent traversals. For example, if a random walk on a network produces the list “dog, cat, hamster, cat, lion,” the censored list would be “dog, cat, hamster, lion”—the second occurrence of “cat” is censored (see Figure 1). This model has been shown to approximate human fluency data in manyways [33, 41]. As previous work focused on healthy individuals, the censoring process was deterministic and did not produce perseverations (repeated items). Repeating items during the semantic fluency task is a hallmark of Alzheimer’s fluency data. To account for perseverations, we modify this process so that data are generated by a noisy censored random walk: repeated items are emitted with some unknown probability *p*_*emit*_ and are censored with probability 1–*p*_*emit*_. When *p*_*emit*_ *=* 0, censoring is deterministic (i.e., no items are ever repeated in the censored random walk) and it is equivalent to the previous model.

Under this model, the probability of a semantic network given a set of *L* fluency lists $X=\left\{X^{1},\ldots ,X^{L}\right\}$ is

(1) $$\begin{array}{l} \mathbb{P}\left(G|X^{1},\ldots ,X^{L},p_{emit}\right)\\ \propto \mathbb{P}(G)\mathbb{P}\left(p_{emit}\right)\prod_{l=1}^{L}\mathbb{P}\left(X^{l}|G,p_{emit}\right) \end{array}$$

where **G** denotes an undirected and unweighted network, and *G*_*ij*_ (for each *i* and *j)* is either 1 or 0 to indicate whether an edge exists between the two concepts associated with indices *i* and *j*. The likelihood of generating any fluency list given a network is the product of all transition probabilities in that list, multiplied by the probability of observing the initial item in that list

(2) $$\begin{array}{l} \mathbb{P}\left(X^{l}|G,p_{\text{emit}}\right)\\ =\mathbb{P}\left(X_{1}^{l}|G\right)\prod_{n=2}^{N_{l}}\mathbb{P}\left(X_{n}^{l}|X_{1}^{l},\ldots ,X_{n-1}^{l},G,p_{emit}\right) \end{array}$$

where $X_{n}^{l}$ denotes the *n*th item of the *l*th fluency list, and *N*_*l*_ denotes the number of items in the *l*th list. We assume that the probability of the initial item in a list is given by the limiting probability of an infinite-length random walk encountering that item’s node (the stationary distribution of a random walk over the network):

(3) $$\mathbb{P}\left(X_{1}^{l}=i|G\right)=\frac{\sum_{m=1}^{M}G_{im}}{\sum_{m=1}^{M}\sum_{p=1}^{M}G_{pm}}$$

where *M* denotes the number of nodes in network **G** (i.e., the total number of unique responses across all lists in *X).* In other words, the probability of an initial item in a list is proportional to the number of edges connected to that item in **G**. In (3) and elsewhere, we use the subscript of an item label and its index within a matrix interchangeably (i.e., if $X_{1}^{l}$ = *i* = “dog”, then *G*_*im*_ indicates whether an edge exists between “dog” and the item label associated with index *m* in **G**).

Each transition probability can be modeled as an absorbing random walk. First, we translate link matrix **G** into a transition probability matrix **A**, where

(4) $$A_{ij}=\frac{G_{ij}}{\sum_{m=1}^{M}G_{im}}$$

We rearrange the rows and columns of **A** to be in list order (the same order as *X*^*l*^), which we denote as **A**^*l*^. Items that do not appear in *X*^*l*^ are excluded from **A**^*l*^. When perseverations occur in *X*^*l*^, only the first occurrence is preserved in **A**^*l*^ (so that each node appears at most once in **A**^*l*^).

For each transition probability $\mathbb{P}\left(X_{n}^{l}|X_{1}^{l},\ldots ,X_{n-1}^{l},G,\;p_{emit}\right)\;$ that is calculated, **A**^*l*^ is decomposed into submatrices:

(5) $$A^{l}=\left[\begin{array}{ll} Q & R\\ 0 & I \end{array}\right]$$

where **Q** denotes transitions between nodes observed prior to the currently considered transition to node *n* (i.e., nodes in $\left\{X_{1}^{l},\ldots ,X_{n-1}^{l}\right\}$) and **R** denotes transitions from previously observed nodes to new nodes (i.e., nodes in $\left\{X_{n}^{l},\ldots ,X_{N_{l}}^{l}\right\}$ ). 0 and **I** denote a matrix of zeroes and the identity matrix, respectively. We then define ***Q*′** as

(6) $$Q^{\prime }=\left(1-p_{emit}\right)\cdot Q$$

and **R**^**′**^ as

(7) $$R^{\prime }=\left[p_{emit}\cdot Q,R\right]$$

**Q′** denotes the probabilities of transitioning from any previously observed node to another previously observed node *while being censored.* **R′** denotes the probabilities of transitioning from any previously observed node to *either* a new node *or* a previously observed node that is not censored. While **Q′** is of the same dimension as **Q′**, **R′** is larger than **R: R′** contains the same number of rows as **R**, but the number of columns in **R′** is equal to the total number of unique items in *X*^*l*^.

We can then calculate a transition probability as

(8) $$\mathbb{P}\left(X_{n}^{l}=i|X_{1}^{l},\ldots ,X_{n-1}^{l}=j,G,p_{emit}\right)\;=\{\begin{array}{ll} \sum_{k=1}^{s}E_{jk}R_{ki}^{\prime } & \;\text{if}\;E\;\text{exists}\;\\ 0 & \;\text{otherwise}\; \end{array}$$

where *s* denotes the number of unique items listed prior to $X_{n}^{l}$ (i.e., the number of rows in **Q′**). **E** is the fundamental matrix of the Markov chain for transition *n* [42]:

(9) $$E={\left(I-Q^{\prime }\right)}^{-1}$$

and *E*_*jk*_ denotes the expected number of times a Markov chain starting at node *j* in transition matrix **Q′** will visit *k* before being absorbed.

We derive the prior probability of a network ℙ(G) using an unweighted and undirected semantic network constructed from the free association norms compiled by the University of South Florida (USF; [43]). These norms were generated by asking over 6,000 participants to respond to a set of cue words with the first meaningfully related word; for instance, if the cue word is “car”, a participant might respond “road”. From these norms, we extracted all animal cue-response pairs (e.g., “dog–cat”) and constructed a semantic network by adjoining each of these pairs with an edge. The network consists of 160 animals and 393 edges.

We assume that the prior probability of an edge in a network is binomial distributed according to whether it occurs in the USF network: $\mathbb{P}\left(G_{ij}=1\right)=2/3$ when an edge exists between *i* and *j* in the USF network, $\mathbb{P}\left(G_{ij}=1\right)=.4$when an edge does not exist in the USF network, and $\mathbb{P}(G_{ij}=\text{1}\;)\;=;\;\text{.5}$ when either *i* or *j* (or both) are not present in the USF network. These free parameters were derived by using a zero-inflated beta-binomial prior, as described in the hierarchical model of Zemla and Austerweil [31], but treating the USF network as the sole, fixed prior network. As such, $\mathbb{P}(G)=\Pi_{i,j}\mathbb{P}\left(G_{ij}\right)$for all *i* and *j* in G. Given the data, we seek to find the network that maximizes the *a posteriori* probability:

(10) $$\underset{G,p_{\text{emit}}}{\arg\max}\mathbb{P}\left(G,p_{emit}|X\right)$$

We do this through stochastic search on the network. We randomly toggle an edge in the network and accept that edge change when the posterior probability of the network after the edge change is greater than the posterior probability of the network before the edge change. We use a set of heuristics to decide which edges to flip and set a tolerance value such that the network “converges” after 300 edge flips that do not increase $\mathbb{P}\left(G,p_{emit}|X\right)$. For further details, see Zemla and Austerweil [31]. After each successful edge toggle, we perform a grid search to find the optimal value for $p_{emit}\in \{0.0,0.01,\ldots ,0.99,1.0\}$ given that network. We assume the prior probability of *p*_*emit*_ is uniformly distributed over these values.

### 2.3. Participant Networks and Mock Networks.

We estimated a semantic network for each participant by diagnosis (NC or PAD) combination in the data set. Each participant had at least three fluency lists available to generate a network. In total, 125 semantic networks were generated: 84 NC networks and 41 PAD networks. This includes two participants who transitioned from healthy to Alzheimer’s diagnosis and had both an NC and PAD network. (More than 2 participants in the dataset converted from NC to PAD, but only 2 participants had a minimum of three NC and three PAD lists required to estimate both networks). The remaining participants (82 NC and 39 PAD) had only one network. PAD networks were generated from an average of 6.05 lists per network (range 3–10), while NC networks were generated from an average of 9.51 lists per network (range 3–26).

We compared PAD networks to NC networks using the following network measures: number of nodes, diameter, density, mean/median node degree, average shortest-path length, clustering coefficient, and small-world coefficient. These measures are further defined in Table 1.

Participants with more fluency lists (and longer fluency lists) will typically have semantic networks that have more nodes and more edges. This is confounding because most network properties (such as diameter or average shortest-path length) are affected by the number of nodes and edges in a network. This makes it difficult to draw inferences from a direct comparison of NC and PAD networks, as the networks vary in the amount of data used to generate them.

To alleviate this problem, we analyzed each network by comparing it to its own set of mock networks generated by the following procedure: For each participant, we generated a random permutation of each fluency list so that the order of the words in each list is arbitrary. We then estimated a network for the set of permuted lists using the same process as their actual network. We repeated this procedure fifty times for each participant. This process ensures that each mock network has the same number of nodes as its corresponding participant network. It also ensures that differences between individuals are not merely due to differences in the distribution of animal frequencies in their lists. Using these mock networks, we can define a distribution of values for any network measure under the assumption that words within a list are arbitrarily ordered. Using this bootstrapping procedure, we can then use standard hypothesis testing techniques to gauge how a participant’s semantic network deviates from other possible networks that could have been inferred (with the exact same amount of fluency data).

## 3. Results and Discussion

### 3.1. Semantic Network Properties and Model Parameters.

**Figure 2:**
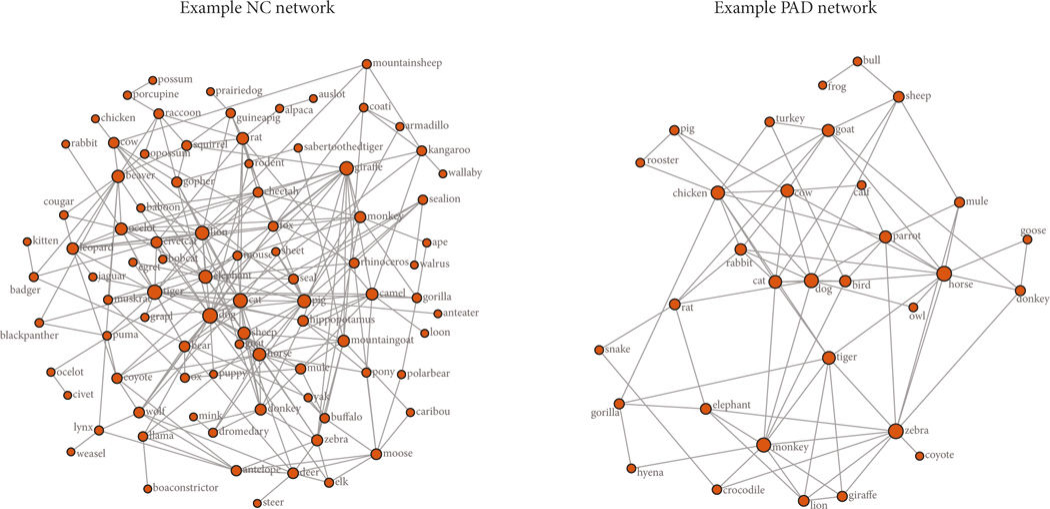
An example network is shown for one NC participant and one PAD participant.

Estimated semantic networks are available as Supplementary Material (available here). An example semantic network for one PAD participant and one NC participant is shown in Figure 2.

PAD patients listed fewer items per list compared to NC patients, *M*_*NC*_ = 19.33, *M*_*PAD*_ = 13.13, *t*(123) = 9.76, *p* < .001, and also had higher rates of perseveration per list, *M*_*NC*_ = .034, *M*_*PAD*_ = .127, *t*(42.9) = 6.92, *p* < .001. We applied a Welch correction here and throughout the paper whenever variances were deemed unequal by an F-test. None of these corrections changes the significance of the test (i.e., they did not affect a decision to reject the null hypothesis).

For descriptive purposes, we present a raw comparison between PAD and NC networks without adjustment using the mock networks. (Many of the factors we examine have some degree of correlation. A full correlation matrix between factors is provided in the Supplementary Material). A summary of the network properties for each network type is shown in Table 2. PAD networks appear different than the NC networks in many ways. On average, NC networks have more nodes than PAD networks, a reflection of the fact that PAD patients list fewer unique animals than NC participants, *M*_*nc*_ = 66.8, *M*_*pad*_ = 32.3, *t*(123.0) = 10.81, *p* < .001. In contrast, PAD networks are denser, *M*_*NC*_ = .060, *M*_*PAD*_ = .116, *t*(47.0) = 7.08, *p* < .001, and have a smaller diameter, *M*_*nc*_ = 9.55, *M*_*pad*_ = 7.61, *t*(123) = 3.34, *p* < .001. The increased density of PAD networks could reflect an increase in the number of spurious associations. This is consistent with previous behavioral findings; for instance, Chan, Butters, Salmon, and McGuire [46] found that while a cohort of AD patients were unimpaired matching animal names to pictures, they tended to group animals into atypical categories.

Perhaps because they are more dense, PAD networks tend to have a shorter average shortest-path length, *M*_*NC*_ *=* 3.74, *M*_*pad*_ *=* 3.25, *t*(123) = 3.24, *p =* .002. NC networks have a higher mean degree, *M*_*NC*_ = 3.55, *M*_*PAD*_ = 3.18, *t*(123) = 2.69, *p* = .008, meaning that healthy control networks have, on average, more semantic associates per concept than AD networks. AD and NC networks do not differ in their median degree (*p* = .18) or in their clustering coefficients (*p* = .15).

A small-world network is one that has a small average shortest-path length but high clustering coefficient [47]. Small-world networks are efficient in that they have low wiring costs (i.e., few edges) but allow fast communication between any two nodes in a network [48]. Previous research has suggested that semantic networks are small-world-like [49]. Small-world networks are commonly seen in language (and other domains), perhaps because they emerge from a simple preferential attachment learning mechanism [49, 50], in which newly learned words are more likely to connect to other high-degree words in an existing semantic network than to low-degree words.

Small-worldness can be quantified as the ratio of a network’s clustering coefficient relative to random network, over the ratio of a network’s average shortest-path length relative to a random network [45]. Networks with a small- world coefficient greater than one are said to be small-world networks. We found that PAD networks are significantly less small-world like compared to NC networks, *M*_*NC*_ = 1.92, *M*_*pad*_ = 1.21, *t*(107.2) = 4.91, *p* < .001, suggesting the efficient interconnectivity of healthy semantic networks is degraded in AD patients.

In addition, PAD patients typically had a higher value for their perseveration parameter *p*_*emit*_*, M*_*NC*_ *=* .07, *M*_*PAD*_ *=* .34, *t*(42.3) = 5.34, *p <* .001. Under the noisy censored random walk framework, this indicates that the internal monitoring process of PAD patients (deciding whether a word has been said previously) is impaired relative to NC participants. This may be expected, given that PAD patients have higher rates of perseveration in the data, though a higher rate of perseverations in the fluency data does not guarantee a higher value for *p*_*emit*_. The reason for this is that the network structure affects the total number of opportunities for a perseveration to occur. For example, in a fully connected network, an uncensored random walk of a fixed length will produce fewer perseverations than an uncensored random walk of the same length on a linear network

### 3.2. Adjusted Network Properties.

While we observed many differences between PAD and NC networks, it is difficult to judge whether these differences are due to the mental representations of the two groups or whether they emerge because PAD networks are, on average, generated from a smaller amount of data than NC networks. We adjust for this by constructing corresponding mock networks for each participant network. As described in the “Participant Networks and Mock Networks” subsection, these networks were constructed by permuting the fluency lists of each participant and generating a new network using U-INVITE. We then compute delta metrics by subtracting a participant network’s measure from the average of the mock networks. For example,

(11) $$\Delta_{aspl}=G_{aspl}-\left(\frac{\sum_{k=1}^{50}D_{aspl}^{k}}{50}\right)$$

where *G*_*aspl*_ denotes the average shortest-path length of participant network *G* and $D_{aspl}^{k}$ denotes the average shortest-path length of mock network *k* yoked to participant network *G*. (Here, we use 50 in the denominator because we generate 50 mock networks for each participant’s network.)

While both NC and PAD networks have a smaller mean degree compared to their mock counterparts, the difference between the mock and participant networks is smaller for PAD networks than for NC networks, *M*_*NC*_ = –0.81, *M*_*PAD*_ = –0.26, *t*(105.4) = 6.39, *p* < .001. The same pattern is true for the networks’ median degree, *M*_*NC*_ = –0.38, *M*_*PAD*_ = –.15, *t*(123) = 2.25, *p* = .026. Both NC and PAD networks have a larger average shortest-path length compared to mock networks, but again PAD networks are significantly closer to their mock counterparts, *M*_*NC*_ = 0.67, *M*_*PAD*_ = 0.36, *t*(123) = 3.66, *p* < .001. NC and PAD networks also have larger diameters than their corresponding mock networks, though PAD networks are closer to their mock networks, *M*_*NC*_ = 2.43, *M*_*PAD*_ = 1.24, *t*(123) = 2.97, *p* = .004. Collectively, these results suggest that, in many ways, PAD networks more closely resemble networks generated from randomly generated (i.e., permuted) fluency lists. In contrast, NC networks are quite distinct from networks estimated from randomly generated lists.

While both NC and PAD networks are less dense and less clustered relative to their mock network counterparts, the delta scores themselves do not differ between groups for either density (*p* = .47) or clustering coefficient (*p* = .91).

We do not provide comparisons for small-worldness or ***p***_*emit*_ adjusted by their mock networks (though their raw values are listed in Table 2). Unlike other network measures, small-worldness is explicitly measured as a ratio relative to the clustering and shortest-path length of a random (Erdös-Renyi) network (see [45]), so no correction is needed. Additionally, ***p***_*emit*_ is not inherently correlated with network size and does not need to be corrected.

### 3.3. Relation between Network Measures and Alzheimer’s Diagnosis.

We used logistic regression to identify associations between network measures and participant diagnosis (NC or PAD) under several different models (interaction terms were excluded to avoid a combinatorial explosion of parameters). In clinical settings, the semantic fluency task is often scored by examining only the total number of responses given and the perseveration rate. In a baseline model, we used these two factors as independent variables along with years of education, which is widely believed to be associated with Alzheimer’s disease [51]. Both number of responses and perseveration rate were significantly associated with diagnosis (***p*** < .001), as was the model as a whole (***p*** < .001, A/C = 52.38, null deviance = 158.2, residual deviance = 44.4). Though PAD participants reliably differ from NC participants in years of education, *t*(60.9) = 2.56, *p* = .013, years of education was not significantly associated with diagnosis in the baseline model(***p*** = .44) after controlling for other factors in the model.

**FIGURE 3:**
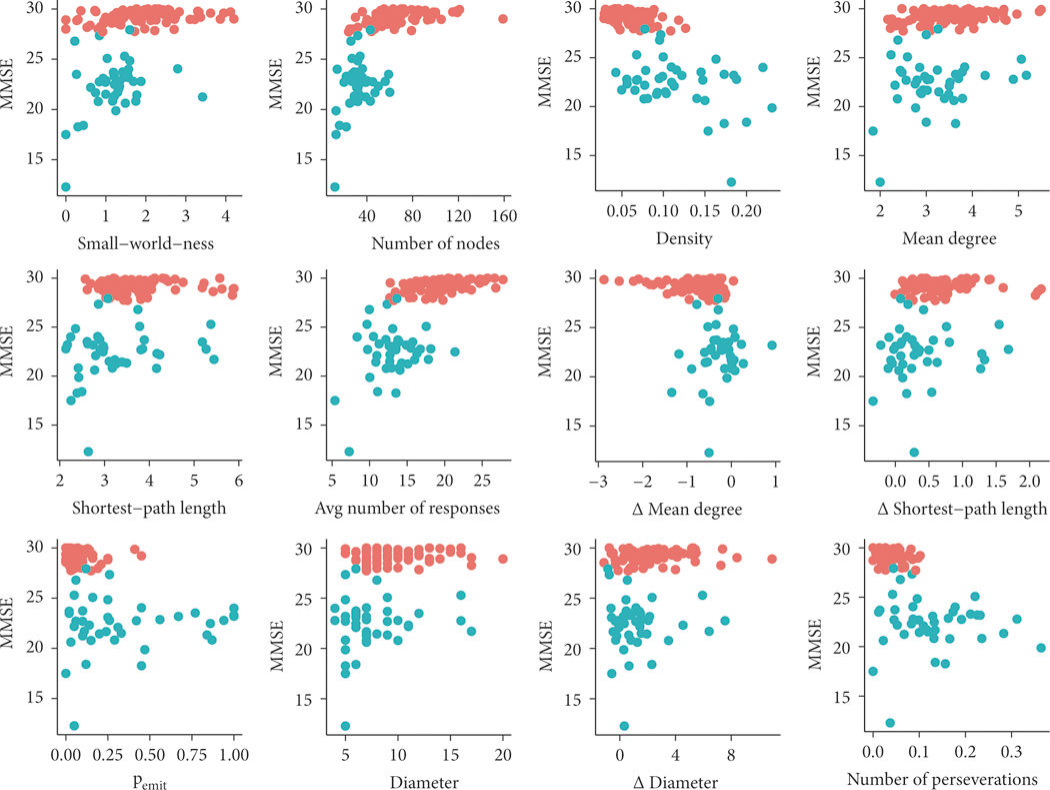
Each of the factors we identified as distinguishing between PAD and NC (except A median degree, correlation *p* = .11) are plotted with respect to scores on the Mini-Mental State Exam (MMSE) (MMSE scores were unavailable for 7 participant visits out of 1,047). Blue dots indicate patients diagnosed with PAD and red dots indicate those diagnosed as NC. Clinicians had access to patient MMSE scores when making their diagnosis, but did not use semantic fluency data to make their diagnoses. All correlations are significant (*p* < .005, uncorrected for multiple comparisons). Many of these correlations appear to be driven by a restriction in range of the MMSE scores for NC patients. Only the first three factors in the top row (small-worldness, number of nodes, and density) are correlated significantly with MMSE (*p* < .05, uncorrected) when restricted to PAD patients.

We compared this baseline model to a maximal model that included ten additional factors. Along with the three factors in the baseline model, we included each of the network measures that are correlated with performance on the Mini-Mental State Exam (i.e., those measures shown in Figure 3: small-worldness, number of nodes, density, mean degree, Δ mean degree, shortest-path length, Δ shortest-path length, *p*_*emit*_, diameter, and Δ diameter.) This maximal model also explained a significant portion of the variance in participant diagnoses (***p*** < .001, A/C = 57.3, residual deviance = 29.3). See Table 3.

We also conducted an exploratory step-wise regression model using bidirectional elimination starting with the maximal model. The best fit model (*p* < .001, A/C = 45.5, residual deviance = 33.5) contained five factors: the total number of responses, perseveration rate, Δ mean degree, *p*_*emit*_, and Δ diameter. Four factors were individually significant (*p* < .05) while one (Δ diameter) was not (*p* = .11). This model outperformed both the baseline and maximal models as measured by AIC, a model selection criterion that penalizes models with more parameters [52].

In addition, we performed a cross-validation of the data to predict the diagnosis of each individual using each of the three models. Cross-validation was performed using split- halves, sampled randomly while preserving the overall ratio of NC and PAD participants in each half (i.e., 67% NC in each training sample). This procedure was repeated on the dataset 5,000 times.

Both the step-wise model and the maximal model outperformed the baseline model in predicting diagnoses using measures of accuracy (average *ACC*_*baseline*_ = 92.5%, *ACC*_*stepwise*_ = 92.9%, *ACC*_*maximal*_ = 96.1%) and F1 scores (average *F*1_*baseline*_ = 883^,^ *F*1_*stepwise*_ = .891^,^ *F*1_*maximal*_ *=* .939). An F1 score denotes the harmonic average of precision and recall. It is used frequently in signal detection analyses to balance the need to correctly predict positive cases and avoid false alarms. It ranges from 0 to 1, where 1 represents perfect precision and recall. A break-down of hits, misses, false alarms, and correct rejections for each model is shown in Table 4. These results suggest that network factors may aid in predicting patient diagnosis. However, because the factors in each model were chosen based on observing the whole dataset, future work is needed to validate these models on an independent dataset.

## 4. Conclusion

Using a longitudinal corpus of semantic fluency data, we estimated animal semantic networks for individuals categorized as either healthy (normal control) or probable Alzheimer’s patients. These networks revealed systematic differences between the mental representations of the two groups. Healthy semantic networks were larger, less dense, and contained a higher number of associations per concept (i.e., higher mean degree). Using a bootstrap approach to generate mock networks, we found that Alzheimer’s networks are significantly closer to mock networks generated from random permutations of the participants’ data. In contrast, healthy networks are more small-world-like, consistent with prior literature on semantic networks [49] and human language [53], and drawing a parallel to a finding that large-scale brain networks in AD patients are also less small-world-like than healthy controls [54].

These results corroborate previous results that have shown atypical associations in the semantic representations of AD patients [46, 55]. Using current best practices for estimating semantic networks, our results corroborate those of Lerner et al. [12] finding that unadjusted AD semantic networks are less dense and less small-world-like, having smaller diameter and higher average node degree.

However those differences between AD and control semantic networks should be interpreted in light of the processes and data used to construct them: using the censored random walk model (or a naïve random walk model used by [12]), smaller data sets (i.e., fewer fluency lists or shorter lists) that are evenly generated at random produce smaller networks (i.e., fewer nodes), which distorts many network properties. In contrast to previous work, we adjusted for this potential confound by comparing each network to a null model (i.e., mock networks) that assumes items in a fluency list do not have any sequential dependencies. This procedure revealed that some findings, such as the difference in the density of networks between groups, may be artifacts of the methodology used to construct networks. Future work should test the robustness of our results against different methods for constructing networks (e.g., [30]) as well as other null models: for example, Chan et al. [5] construct networks using triadic comparison data so that all participant networks have the same number of nodes, while Kenett et al. [56] compare estimated networks to Erdös-Rényi random networks of the same size. The field has yet to come to a consensus on the most appropriate null model to use for comparing networks, though it is likely that each of these approaches have strengths and weaknesses.

Previous attempts at mapping semantic memory in patients with semantic impairments have been criticized as being methodologically inadequate [15]. Part of this criticism stems from constructing group networks by averaging across the representations of individuals, who likely have unique impairments. In contrast to previous research [5, 12], our study is the first to estimate semantic network representations of individual patients with AD. In addition, Verheyen et al. [16] suggest that previous methods used to map semantic representations (such as multidimensional scaling or singular value decomposition; see [57]) do not produce stable representations, even when generated from random samples from the same individual’s data. While this is a concern, our method of generating semantic networks is both theoretically and mathematically distinct from these criticized approaches. The work they criticized used estimation techniques that were exchangeable, which means that the item order in a list did not affect the estimated representation. Our method is nonexchangeable. Changing the order of items in a list affects the probability of estimated networks due to earlier items being much less likely to be affected by censoring than later items. Further, Zemla and Austerweil [31] found that the deterministic censoring version of our method for estimating networks from fluency data was empirically valid: estimated edges were judged to have high semantic similarity in pairwise similarity ratings compared to nonedges.

Similarly, Verheyen et al. [16] suggest that perhaps semantic fluency data cannot reliably estimate semantic representations because the semantic fluency task taps into other cognitive processes in addition to representation. We agree, and we caution using our results to advocate for a purely storage deficit (as opposed to retrieval deficit) in AD. The censored random walk model of memory retrieval on which our network inference method is based [31] assumes that semantic retrieval is biased towards items that are semantically similar to recently retrieve items. While this is generally accepted to be true, mental search may also rely on frontal lobe processes that are independent of the semantic representation [58]. Future work may modify the censored random walk model that allow for random or strategic jumps [33, 41] that reflect an explicit cluster switching component (or “restarts” of the search process) and possibly distinguish the influence of representation versus executive functioning on mental search. Though previous work suggests these jumps are not necessary to model healthy fluency behavior, they may play a role when modeling behavior from populations with memory disorders. Furthermore, we find differences in a working memory monitoring process of NC and AD patients (as evidenced by differences in *p*_*emit*_) that more closely align with retrieval rather than storage deficits. It is likely that the semantic impairments of AD patients are due in part to both storage and retrieval deficits, and our results suggest one model that explains these impairments through an interaction of the two.

One limitation of our current approach is that we only look at the structure of semantic networks in the animal category. Although the animal fluency task is extremely common in the psychology literature and in clinical practice, it may not be the best proxy for an individual’s semantic memory as a whole. Chan, Salmon, and De La Pena [59] found that while semantic representations of the animal category are impaired in AD, the tools category remained largely intact. Though a focus on the animal category may be useful for identifying semantic decline in AD patients, analysis of a broader spectrum of categories could provide a more wholistic view of semantic memory impairment in AD.

Finally, we found that the inclusion of network measures in a logistic regression model improved prediction of participant diagnosis, even after adjusting for the increased number of parameters. This suggests that a network-based approach may explain more variance than traditional approaches to scoring the semantic fluency task and could improve identification of patients with Alzheimer’s disease.

The current research highlights potential differences in the structural properties of semantic networks of individuals with and without Alzheimer’s disease, yet raises additional questions about the computational processes that might produce these changes. As patients transition from healthy to impaired, are changes to their semantic network better modeled by the addition or removal of edges, or some combination of both? Are edged removed (or added) at random in the network, or do these changes occur at predictable locations in the network? For example, are edge changes more likely at high-degree nodes? Do they spread from “infected” nodes? Our current results suggest that a process that adds spurious edges at random might be a good candidate for exploration, but further research is needed.

In the above analyses, we consider only two diagnostic points: healthy (NC) and probable Alzheimer’s diagnoses (PAD). Future research should examine individuals with Mild Cognitive Impairment, an intermediary phase between healthy and Alzheimer’s disease, as well as track the networks of individuals as they evolve over time. In doing so, it may be possible to uncover the dynamic processes that explain the transition between healthy and impaired networks. While biological models of how AD spreads have been postulated (e.g., [60]), no such processes have been proposed on the algorithmic level for semantic network degradation.

We believe our results represent the first attempt to estimate individual semantic networks from a psychologically plausible process model in order to assess memory impairment. Future research can extend this approach in many ways. Many clinical populations other than Alzheimer’s patients are impaired on the semantic fluency task—including those with Huntington’s disease [61], frontotemporal dementia [62], and semantic dementia [63]. Studies have found that these groups may have distinct behavioral profiles on the semantic fluency task, and perhaps their semantic networks are distinct as well.

Overall, we find that a network-based analysis of semantic fluency data may improve diagnosticity of Alzheimer’s disease, while providing clues to the cognitive mechanisms that lead to impairment on the semantic fluency task. This approach may provide a useful tool for assessing other neuropsychiatric disorders and provide new insight into how we store and retrieve semantic knowledge.

## Supplementary Material

2

## Figures and Tables

**TABLE 1:** Network measures.

Measure	Definition

Number of nodes	The total number of nodes in a network
Diameter	The longest shortest-path between any two nodes in a network
Density	A ratio of the number of edges in a network compared to the total number of possible edges in that network
Average shortest-path length	The average length of the shortest-paths between all pairs of nodes
Clustering coefficient	A measure of a network’s tendency for a node’s neighbors to be connected to each other, defined as 3 times the number of triangles over the number of connected triplets [44]
Small-world coefficient	A measure of a network’s “small-worldness” [45]. A small-world network refers to one that has a high clustering coefficient but low average shortest-path length
Node degree	The number of edges connected to a node. Mean degree is the average of every node’s degree in a network

**TABLE 2:** Summary statistics for both PAD an NC networks, as well as mock PAD and NC networks. A dashed line indicates no difference between the mock networks and nonmock statistic. Average shortest-path length and diameter were computed on the largest component of each network, as they are undefined on networks with multiple components.

	NC	NC_*mock*_	PAD	PAD_*mock*_

Number of networks	84	—	41	—
Number of lists	9.51	—	6.05	—
Number of items listed	19.3	—	13.1	—
Number of nodes	66.8	—	32.3	—
Mini-mental state exam (MMSE)	29.2	—	22.4	—
Diameter	9.55	7.12	7.61	6.37
Density	.06	.07	.12	.13
Mean node degree	3.55	4.37	3.18	3.44
Median node degree	2.55	2.93	2.37	2.51
Average shortest-path length	3.74	3.07	3.25	2.89
Clustering coefficient	.12	.15	.14	.17
Perseveration rate	.034	—	.127	—
Perseveration parameter *(p*_*emit*_*)*	.071	.097	.345	.304
Small-world coefficient	1.92	2.05	1.21	1.36

**TABLE 3:** Comparison of logistic regression models.

Baseline model (*AIC* = 52.4)			Maximal model (*AIC* = 57.3)			Stepwise model (*AIC* = 45.5)		
Factor	*z*-value	*p*-value	Factor	*z*-value	*p*-value	Factor	*z*-value	*p*-value

Num responses	4.31	< .001*	Num responses	2.39	< .017*	Num responses	3.17	.002^*^
Perseveration rate	3.71	< .001*	Perseveration rate	1.34	.18	Perseveration rate	1.98	.047^*^
Education	.77	.44	Education	1.07	.29			
			*P*_*emit*_	2.06	.039*	*P*_*emit*_	2.11	.035^*^
			Δ Mean degree	1.58	.11	Δ Mean degree	2.21	.027^*^
			Δ Diameter	1.08	.28	Δ Diameter	1.59	.11
			Diameter	.88	.38			
			Mean degree	1.26	.21			
			Density	.06	.95			
			Shortest-path length	.80	.42			
			A Shortest-path length	.73	.47			
			Small-worldness	.66	.51			
			Num nodes	.56	.58			

**TABLE 4:** The hits, misses, false alarms, and correct rejections for each model are shown below, averaged across 5,000 split halves.

	Baseline	Stepwise	Maximal

Hits	36.02	36.67	38.11
Misses	5.02	4.37	2.93
False alarms	4.49	4.51	2.00
Correct rejections	80.49	80.47	82.98
