# Supplementary material for: Analyzing Knowledge Retrieval Impairments Associated with Alzheimer’s Disease Using Network Analyses
Source: Complexity. Author manuscript; Available in PMC 2019 Jul 24. (PMC6656530; doi:10.1155/2019/4203158)

Below are the correlations between factors listed in Table 2. Those boxes marked with an "X" are those that are not significant (p > .05).

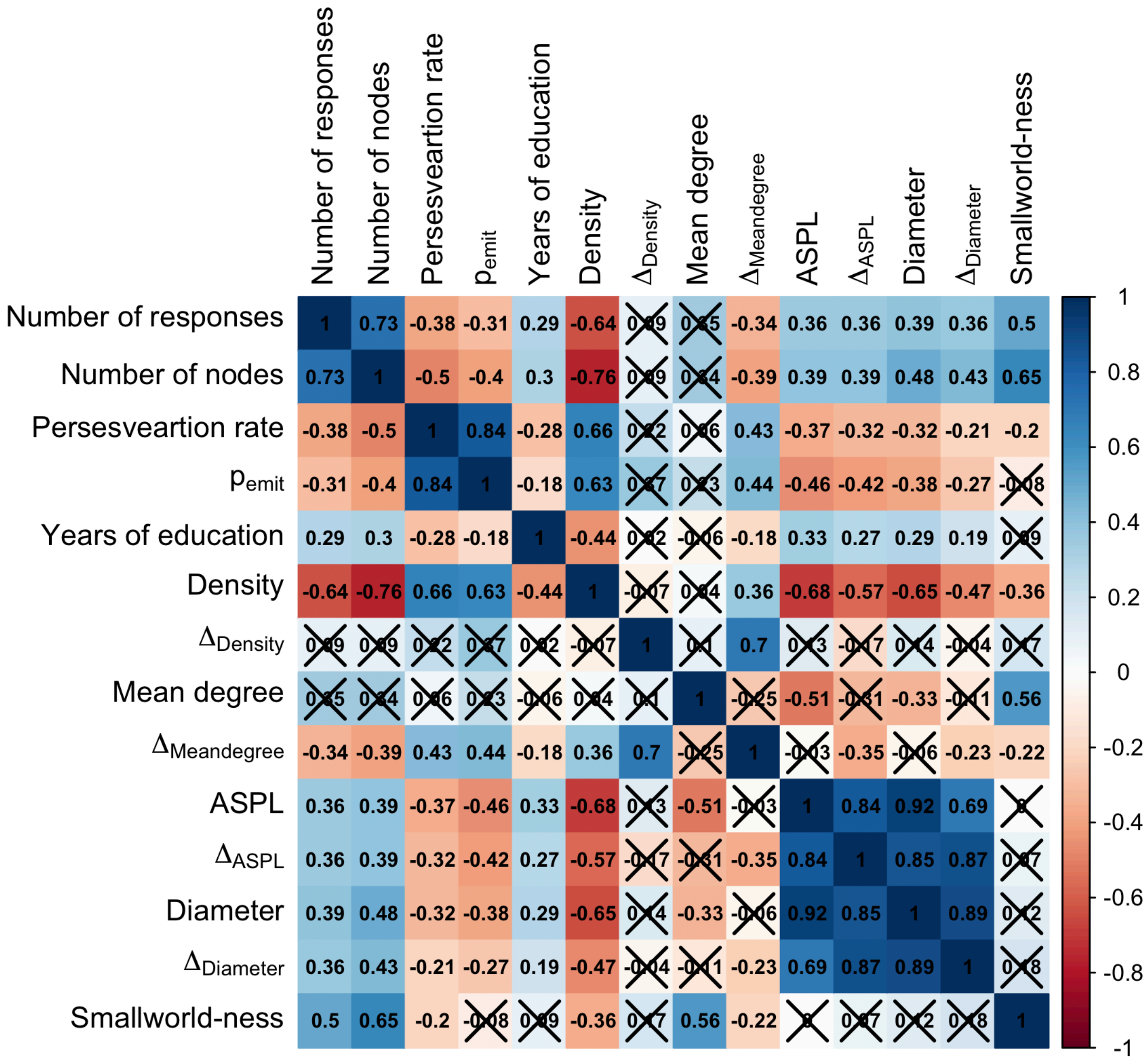

Supplement: 2 [file NIHMS1027540-supplement-2.zip › correlation_matrix.pdf]
